# The fragmented mitochondrial genomes of two *Linognathus* lice reveal active minichromosomal recombination and recombination hotspots

**DOI:** 10.1016/j.isci.2023.107351

**Published:** 2023-07-16

**Authors:** Yi-Tian Fu, Renfu Shao, Suleman, Wei Wang, Hui-Mei Wang, Guo-Hua Liu

**Affiliations:** 1 Research Center for Parasites & Vectors, College of Veterinary Medicine, Hunan Agricultural University, Changsha 410128, China; 2 Centre for Bioinnovation, School of Science, Technology and Engineering, University of the Sunshine Coast, Maroochydore, QLD 4556, Australia; 3 Department of Zoology, University of Swabi, Khyber Pakhtunkhwa 23430, Pakistan

**Keywords:** Entomology, Phylogenetics, Genomics

## Abstract

Evidence for recombination between mitochondrial (mt) minichromosomes has been reported in sucking lice, but it is still not clear how frequent mt minichromosomal recombination occurs. We sequenced the mt genomes of the cattle louse *Linognathus vituli* and the goat louse *L. africanus.* Both *Linognathus* species have 10 mt minichromosomes, and seven of them have the same gene content and gene arrangement. Comparison of mt karyotypes revealed numerous inter-minichromosomal recombination events in the evolution of *Linognathus* species. Minichromosome merger, gene duplication and gene translocation occurred in the lineage leading to *Linognathus* lice. After the divergence of *L. vituli* and *L. africanus*, duplication, degeneration, deletion and translocation of genes also occurred independently in each species. Most of the recombination events in the *Linognathus* species occurred upstream of either *cox3* or *nad2*, indicating these two locations were hotspots for inter-minichromosomal recombination. Our results provide an important perspective on mt genome evolution in metazoans.

## Highlights

• Lice in the genus *Linognathus* have fragmented mitochondrial genomes
• Numerous inter-minichromosomal recombination events occurred in *Linognathus* species
• Minichromosomal locations upstream of *cox3* and *nad2* are hotspots for recombination

## Graphical abstract

**Figure:**
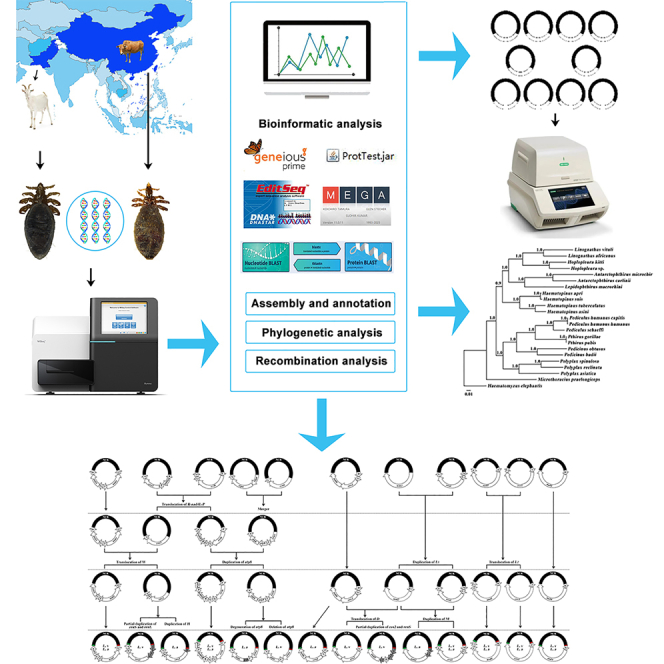

## Introduction

Mitochondrial (mt) genomes have been extensively explored in evolutionary studies of metazoans due to their high mutation rates and maternal inheritance of these genomes.^1^ Metazoan mt genomes typically have a single chromosome with 36–37 genes (including 12–13 protein-coding genes, 22 tRNA genes, and two rRNA genes), a single large non-coding region (NCR), and often highly conserved gene arrangement.^1^ Sucking lice (Anoplura), however, have an unusual, fragmented mt genome organization. These lice feed on host blood and are of medical and veterinary importance as ectoparasites and vectors of disease-causing microorganisms.^2^^,^^3^ An extremely fragmented mt genome with 20 minichromosomes was found first in the human body louse, *Pediculus humanus humanus*.^4^^,^^5^ To date, 21 species of sucking lice from eight families have been sequenced; all these species have fragmented mt genomes with nine to 20 minichromosomes in each species.^4^^,^^5^^,^^6^^,^^7^^,^^8^^,^^9^^,^^10^^,^^11^^,^^12^^,^^13^^,^^14^^,^^15^^,^^16^ The mt karyotypes, i.e., number of minichromosomes, gene content and gene arrangement in each minichromosome,^17^ vary substantially between families, between genera, and even between congeneric species of sucking lice.^5^^,^^12^^,^^13^^,^^16^^,^^17^

Recombination between mt minichromosomes has been proposed as one of the mechanisms that lead to the highly variable mt karyotypes among sucking lice.^9^ Shao et al., showed stretches of identical sequences shared between mt genes on different minichromosomes as unequivocal evidence for inter-minichromosomal recombination.^4^^,^^5^ This type of evidence has been found in 13 species of sucking lice; the shared stretches of identical sequences are up to 133 bp long and are much longer than expected by chance.^4^^,^^5^^,^^6^^,^^8^^,^^9^^,^^10^^,^^11^^,^^12^^,^^14^^,^^16^ Furthermore, Shao and Barker reported eight types of chimeric mt minichromosomes in the human body louse and presented evidence for both homologous recombination and non-homologous recombination between mt minichromosomes in this louse.^18^ Inter-minichromosomal recombination explains well the highly variable mt karyotypes observed in sucking lice, in particular gene translocation between different minichromosomes and merger of minichromosomes.^9^^,^^11^^,^^12^^,^^14^^,^^16^ It is still not clear, however, how frequent mt minichromosomal recombination occurs in sucking lice, and whether recombination tends to occur more often at any particular minichromosomal locations.

The mt genome organizations of sucking lice in seven families (Enderleinellidae, Linognathidae, Hamophthiriidae, Hybophthiridae, Neolinognathidae, Pecaroecidae, and Ratemiidae) remain unstudied. In the current study, we sequenced the complete mt genomes of two *Linognathus* species from the family Linognathidae. *Linognathus* species are among the most common ectoparasites of ruminants,^19^ causing major economic losses to livestock industry worldwide.^20^ We compared the mt karyotypes between these two *Linognathus* species and with the inferred ancestral mt karyotype of sucking lice to further understand inter-minichromosomal recombination in sucking lice.

## Results

### Mitochondrial genomes of the cattle louse *L. vituli* and goat louse *L. africanus*

**Figure 1:**
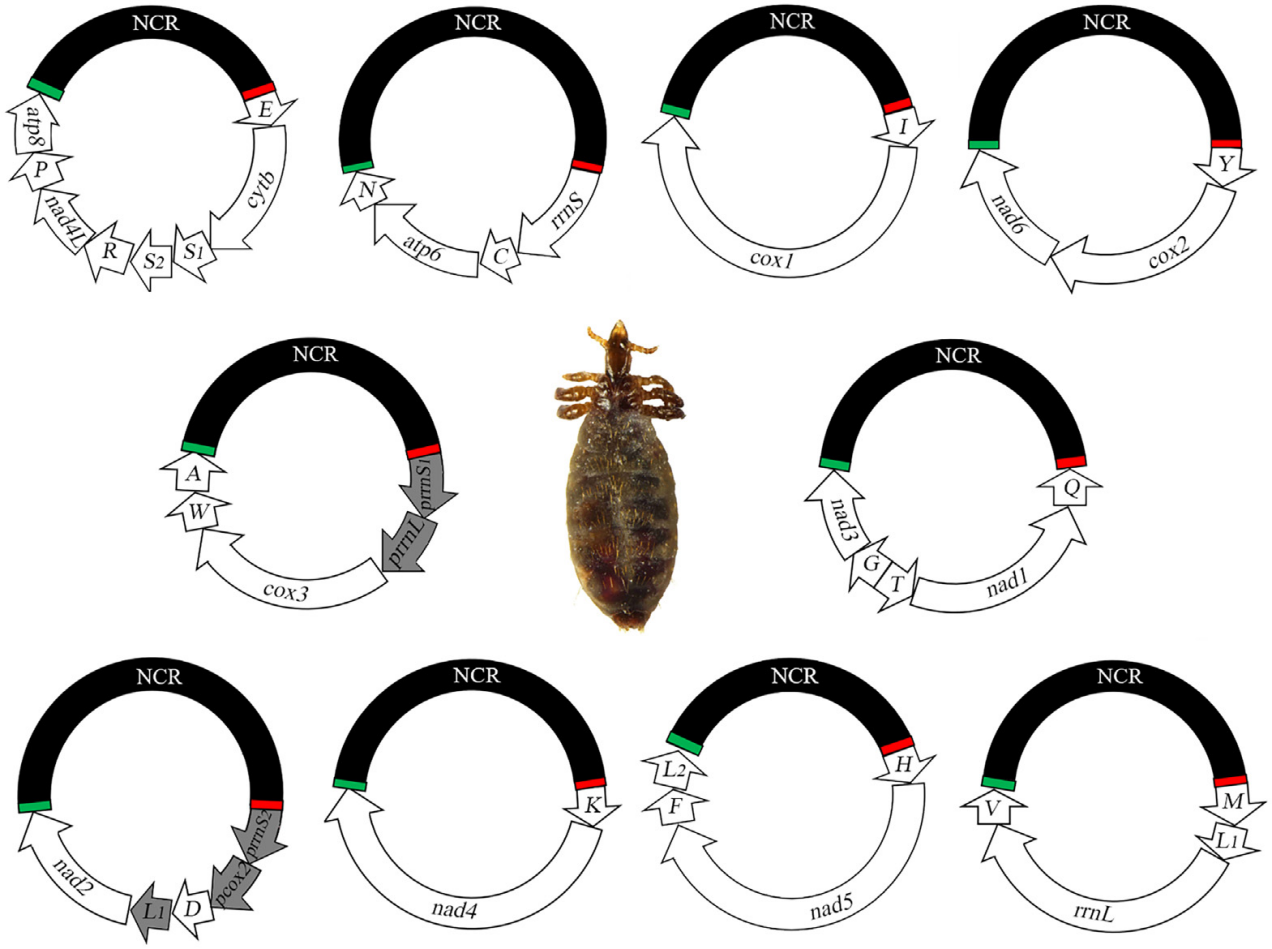
The mitochondrial genome of the cattle louse *Linognathus vituli* Each minichromosome has a region with a cluster of genes and a large non-coding region (NCR). Genes are: *atp6* and *atp8* for ATP synthase F0 subunits 6 and 8; *cytb* for cytochrome *b*; *cox1*-*3* for cytochrome *c* oxidase subunits 1–3; *nad1*-*6* and *nad4L* for NADH dehydrogenase subunits 1–6 and 4L; *rrnS*, and *rrnL* for small and large subunits of ribosomal RNA. tRNA genes are indicated with their single-letter abbreviations of the corresponding amino acids. Arrows indicate orientation of gene transcription. The duplicate *trnL*_*1*_ gene and the four pseudo genes are shaded in gray. Large NCRs are shaded in black. The original copy of *trnL*_*1*_ gene is upstream of *rrnL* gene. Green bars indicate a conserved AT-rich motif (45 bp, 100% A and T) in the large NCRs. Red bars indicate a conserved GC-rich motif (78 bp, 60% C and G) in the large NCRs.

We obtained a large number of clean reads for *L. vituli* using Miseq paired-end sequencing: 6,894,874 pairs from the genomic DNA, and 158,040,342 pairs from the PCR amplicons of individual minichromosomes (Table 1). We assembled these sequence-reads into contigs and identified the typical 37 mt genes in *L. vituli* (13 protein-coding genes, 22 tRNA genes, and two rRNA genes), and a duplicate *trnL*_*1*_ gene, two pseudo *rrnS* genes, a pseudo *rrnL* gene, and a pseudo *cox2* gene (Figure 1; Table 1). These genes and pseudo genes are on 10 minichromosomes; each minichromosome is 2,086 to 3,052 bp in size and consists of a region with genes and a large NCR in a circular organization (Figure 1; Table 1). Each minichromosome has two to eight genes clustered in a region, 893 bp to 1,887 bp in size (Table 1). All genes are transcribed in the same direction except for *trnT-nad1-trnQ* gene cluster (genes underlined have opposite orientation of transcription to other genes) (Figure 1). We sequenced the full-length large NCRs of all of the 10 mt minichromosomes of *L. vituli*, which were 686 bp to 1,296 bp in size (Table 1). A conserved AT-rich motif (45 bp, 100% A and T) is present in every minichromosome in the large NCR upstream of the gene cluster, and a conserved GC-rich motif (78 bp, 60% C and G) is downstream of the gene cluster (Figure 1). The nucleotide sequences of the mt minichromosomes of *L. vituli* have been deposited in GenBank under accession numbers OL677823-32.

**Table 1:** Mitochondrial minichromosomes of the cattle louse *Linognathus vituli*, identified by Illumina sequencing

Minichromosome	Size (bp)	Size of the region with genes (bp)	Size of the large non-coding region (bp)	Size of pseudo genes (bp)	Number of Illumina sequence-reads
*E-cytb-S*_*1*_*-S*_*2*_*-R-nad4L-P-atp8*	3,052	1,887	1,160	0	10,583,104
*rrnS-C-atp6-N*	2,656	1,473	1,179	0	5,042,666
*I-cox1*	2,752	1,566	1,151	0	4,935,026
*Y-cox2-nad6*	2,369	1,218	1,141	0	22,420,706
*prrnS*_*1*_*-prrnL-cox3-W-A*	2,086	893	686	435	4,467,266
*Q-nad1-T-G-nad3*	2,722	1,417	1,296	0	26,553,522
*prrnS*_*2*_*-pcox2-D-L*_*1*_*-nad2*	2,709	1,133	1,112	416	13,987,566
*K-nad4*	2,469	1,286	1,182	0	20,764,008
*H-nad5-F-L*_*2*_	3,000	1,830	1,170	0	3,722,896
*M-L*_*1*_*-rrnL-V*	2,443	1,310	1,133	0	24,359,402
Total	26,258	14,013	11,210	851	158,040,342

**Figure 2:**
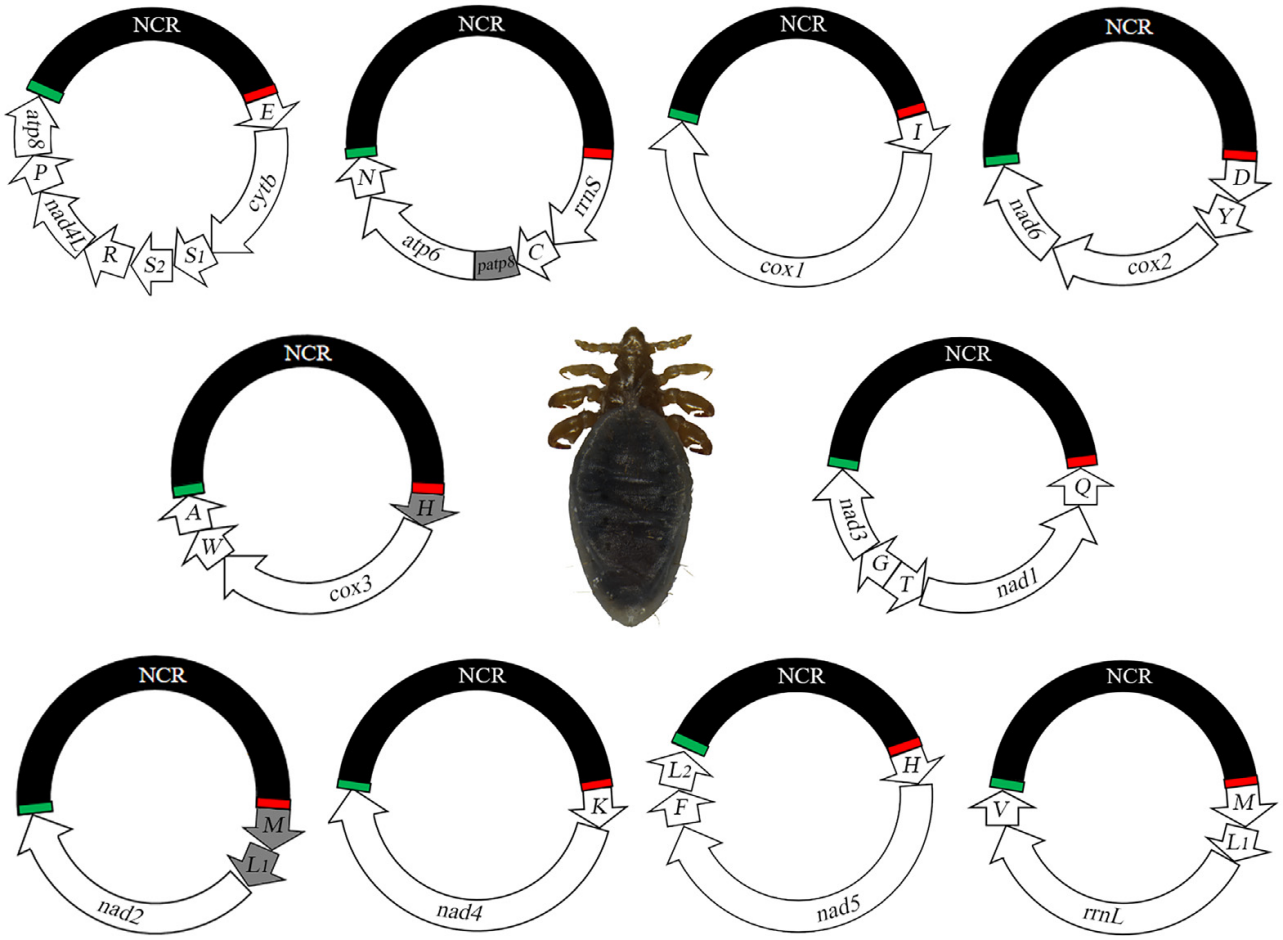
The mitochondrial (mt) genome of the goat louse *Linognathus africanus* See Figure 1 legend for information of mt minichromosomes, genes and large non-coding regions (NCR). Duplicate genes and pseudo *atp8* gene are shaded in gray. Large NCRs are shaded in black. The original copy of *trnL*_*1*_ and *trnM* are upstream of *rrnL*. The original copy of *trnH* is upstream of *nad5*. Green bars indicate a conserved AT-rich motif (60 bp, 75% A and T) in the large NCRs. Red bars indicate a conserved GC-rich motif (38 bp, 76.3% C and G) in the large NCRs.

We obtained 7,378,362 pairs of clean reads from the genomic DNA and 194,422,436 pairs of clean reads from the PCR amplicons of individual minichromosomes of *L. africanus* using Miseq paired-end sequencing (Table 2). Additional to the typical 37 mt genes, three duplicate tRNA genes (*trnL*_*1*_, *trnH*, *and trnM*) and a pseudo *atp8* gene were also identified in *L. africanus* (Figure 2; Table 2). These genes and pseudo gene are on 10 circular minichromosomes, 3,102 bp to 3,915 bp in size (Figure 2; Table 2). Each minichromosome has two to eight genes clustered in a region, 965 bp to 1,874 bp in size (Table 2). All genes are transcribed in the same direction except for *trnT-nad1-trnQ* gene cluster (Figure 2). The large NCRs of *L. africanus* were 1,892 bp to 2,519 bp in size (Table 2). A conserved AT-rich motif (60 bp, 75% A and T) is present in every large NCR upstream of the gene cluster and a conserved GC-rich motif (38 bp, 76.3% C and G) is downstream of the gene cluster (Figure 2). The nucleotide sequences of the mt minichromosomes of *L. africanus* were deposited in GenBank under accession numbers OP948897-906.

**Table 2:** Mitochondrial minichromosomes of the goat louse *Linognathus africanus*, identified by Illumina sequencing

Minichromosome	Size (bp)	Size of the region with genes (bp)	Size of the large non-coding region (bp)	Size of pseudo genes (bp)	Number of Illumina sequence-reads
*E-cytb-S*_*1*_*-S*_*2*_*-R-nad4L-P-atp8*	3,769	1,874	1,892	0	10,857,759
*rrnS-C-patp8-atp6-N*	3,653	1,470	2,011	170	22,987,775
*I-cox1*	3,725	1,599	2,126	0	19,462,215
*D-Y-cox2-nad6*	3,333	1,303	2,026	0	18,751,021
*H-cox3-W-A*	3,486	965	2,519	0	26,785,024
*Q-nad1-T-G-nad3*	3,371	1,409	1,944	0	21,569,810
*M-L*_*1*_*-nad2*	3,102	1,147	1,940	0	13,820,036
*K-nad4*	3,472	1,266	2,170	0	13,145,296
*H-nad5-F-L*_*2*_	3,915	1,829	2,085	0	27,784,003
*M-L*_*1*_*-rrnL-V*	3,444	1,295	2,149	0	24,025,412
Total	35,270	14,193	20,862	170	194,422,436

### Duplicate genes and pseudo genes in the mitochondrial genomes of *Linognathus* species

**Figure 3:**
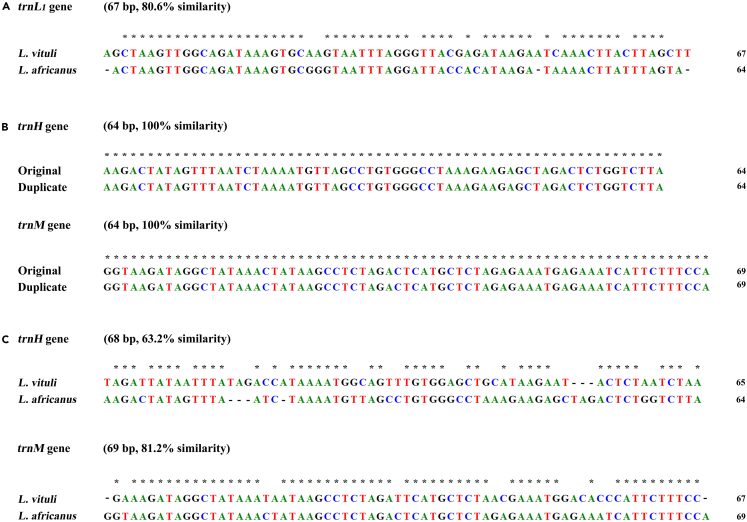
Sequence alignments, lengths and similarities of original and duplicate tRNA genes in the two *Linognathus* lice (A) Sequence alignments, lengths, and similarities of the two copies of *trnL*_*1*_ gene between the two *Linognathus* lice. (B) Sequence alignments, lengths, and similarities of the two duplicate tRNA (*trnH* and *trnM*) genes with their corresponding original tRNA genes in the goat louse *L. africanus*. (C) Sequence alignments, lengths, and similarities of the two copies of *trnH* and *trnM* genes between the two *Linognathus* lice. tRNA genes are indicated with their single-letter abbreviations of the corresponding amino acids.

Four duplicate tRNA genes and five pseudo genes in total were found in the mt genomes of *L. vituli* and *L. africanus*. All of the duplicate genes and pseudo genes are located in different minichromsomes from their corresponding original gene copies or the full-length genes (Figures 1 and 2). In both species, the original copy of *trnL*_*1*_ is retained at its inferred ancestral location upstream of *rrnL* whereas a duplicate *trnL*_*1*_ is present in a different minichromosome upstream of *nad2* (Figures 1 and 2). The two copies of *trnL*_*1*_ of each *Linognathus* species are identical in sequence and length, while between the two *Linognathus* species their *trnL*_*1*_ genes differ by 19.4% in sequence (Figure 3A). Similarly, the other two duplicate tRNA genes (*trnH* and *trnM*) of *L. africanus* also have identical sequences and lengths as their corresponding original copy of tRNA genes (Figure 3B), which are retained at their inferred ancestral locations on different minichromosomes (Figure 2). Between the two *Linognathus* species, their *trnH* genes differ by 36.8% and their *trnM* genes differ by 18.8% (Figure 3C).

There are two pseudo *rrnS* genes, a pseudo *rrnL* gene and a pseudo *cox2* gene in *L. vituli* (Figure 1), and a pseudo *atp8* gene in *L. africanus* (Figure 2). The pseudo genes of *L. vituli* are 198 bp to 252 bp in size and are much shorter than their corresponding full-length genes (Figures S1–S3). The pseudo genes of *L. vituli*, however, are identical in sequence or 99.5% similar to a portion of their corresponding full-length genes (Figures S1–S3). In contrast, the pseudo *atp8* gene of *L. africanus* is very similar in length (7 bp shorter) to the full-length *atp8* but has much lower nucleotide sequence similarity (44.6%) with the full-length *atp8* (Figure S4). The pseudo *atp8* gene of *L. africanus* does not have a start codon, nor a stop codon, nor the highly conserved sequence motif at the 5′ end for MPQ amino acid sequence (Figures S4 and S5). In both *Linognathus* species, *atp8* is on a minichromosome with *cytb*, *nad4L*, and five tRNA genes (Figures 1 and 2). The pseudo *atp8* of *L. africanus* is upstream of *atp6* in the inferred ancestral location of *atp8* (Figure 2); no pseudo *atp8* gene can be found in *L. vituli* (Figure 1).

### Variation in mitochondrial karyotype between *L. vituli* and *L. africanus*

*Linognathus vituli* and *L. africanus* are distinct from each other in mt karyotype although both species have 10 minichromosomes (Figures 1 and 2). Seven of their 10 minichromosomes have the same gene content and gene arrangement between the two *Linognathus* species; the other three minichromosomes, however, differ in gene content and gene arrangement between the two species. In *L.* *vituli*, one of the minichromosomes has three genes in a cluster, *trnY-cox2-nad6*; in *L. africanus*, however, the minichromosome that has this gene cluster also has *trnD* upstream of *trnY* (Figures 1 and 2). Another minichromosome of *L. vituli* has three genes, a pseudo *rrnS* and a pseudo *rrnL*, i.e., *prrnS*_*1*_*-prrnL-cox3-trnW-trnA*. In *L.* *africanus*, however, the minichromosome that has *cox3-trnW-trnA* gene cluster does not have any pseudo genes but has a duplicate *trnH* upstream of *cox3* (Figures 1 and 2). Furthermore, in *L. vituli*, one minichromosome has three genes, a pseudo *rrnS* and a pseudo *cox2*, i.e., *prrnS*_*2*_*-pcox2-trnD-trnL*_*1*_*-nad2*. In *L.* *africanus*, however, the corresponding minichromosome has only three genes, *trnM-trnL*_*1*_*-nad2* (Figures 1 and 2).

### Phylogenetic relationships of *Linognathus* species to other sucking lice

**Figure 4:**
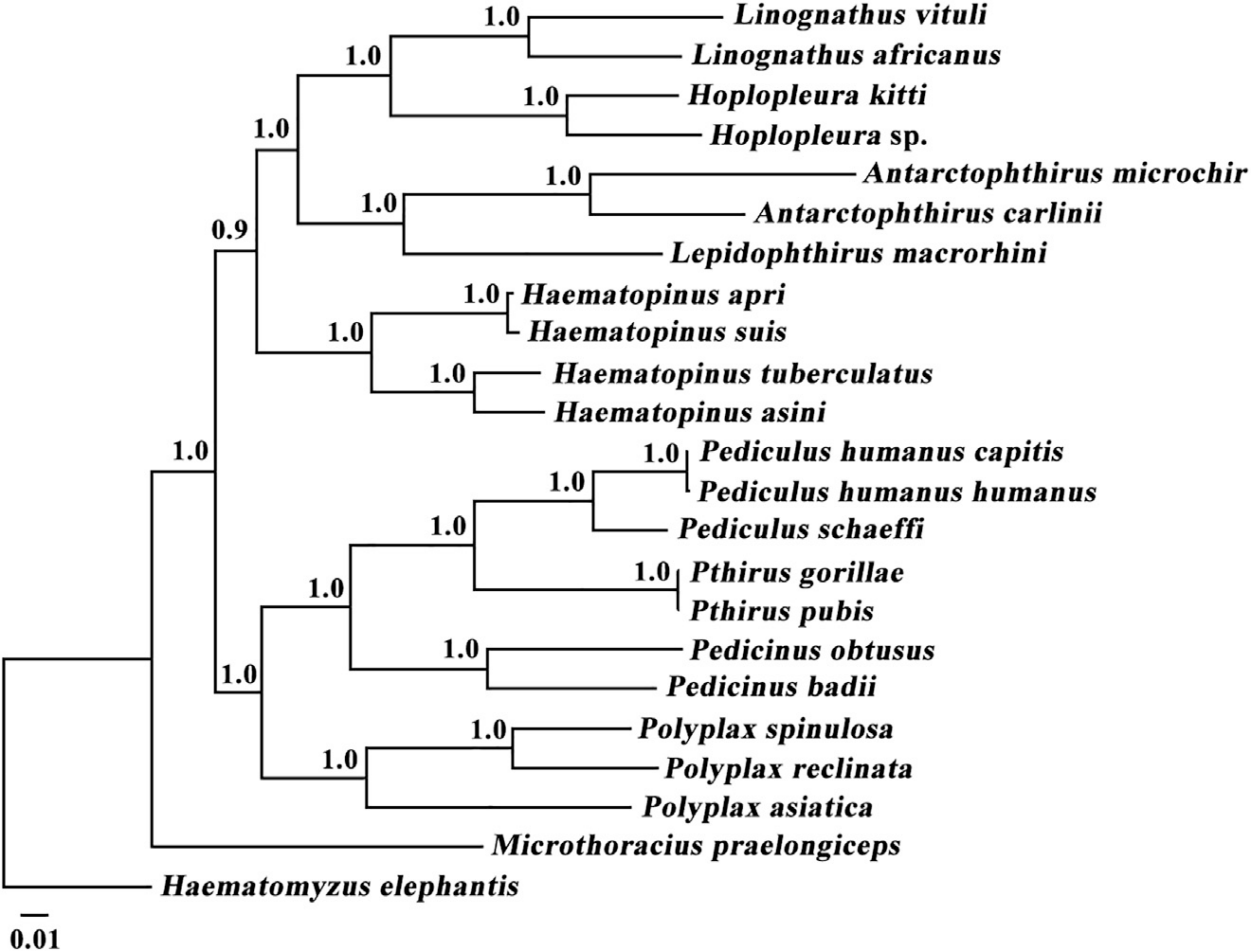
Phylogenetic relationships among 21 species of sucking lice inferred by Bayesian method of deduced amino acid sequences of eight mitochondrial proteins The elephant louse, *Haematomyzus elephantis*, was used as the outgroup. Bayesian posterior probabilities are indicated at nodes.

*Linognathus vituli* and *L. africanus* are most closely related to one another among the 21 species of sucking lice included in our phylogenetic analyses with strong support (Bayesian posterior probability [Bpp] 1.0, maximum-likelihood [ML] bootstrap value [Bv] 100%; Figures 4 and S6). The *Linognathus* species and the rodent lice in the genus *Hoplopleura* are most closely related and form a well-supported monophyletic group (Bpp 1.0, Bv 100%). Four other well-supported monophyletic groups were also identified in both Bayesian and ML trees (Bpp 1.0, Bv 100%; Figures 4 and S6). These groups are: (1) seal lice in the genera *Antarctophthirus* and *Lepidophthirus*; (2) lice of pigs, buffalo, and horse in the genus *Haematopinus*; (3) higher primate lice in the genera *Pediculus*, *Pthirus*, and *Pedicinus*; and (4) rodent lice of the genus *Polyplax*. The guanaco louse, *Microthoracius praelongiceps*, is on its own branch, not in any of the five monophyletic groups. The relationships among the five monophyletic groups and the guanaco louse are well resolved in the Bayesian tree (Figure 4) but are not resolved in the ML tree with weak support values (Figure S6).

## Discussion

### *Linognathus* species are distinct in mitochondrial karyotype from other sucking lice and the inferred most recent common ancestor of sucking lice (Anoplura)

**Figure 5:**
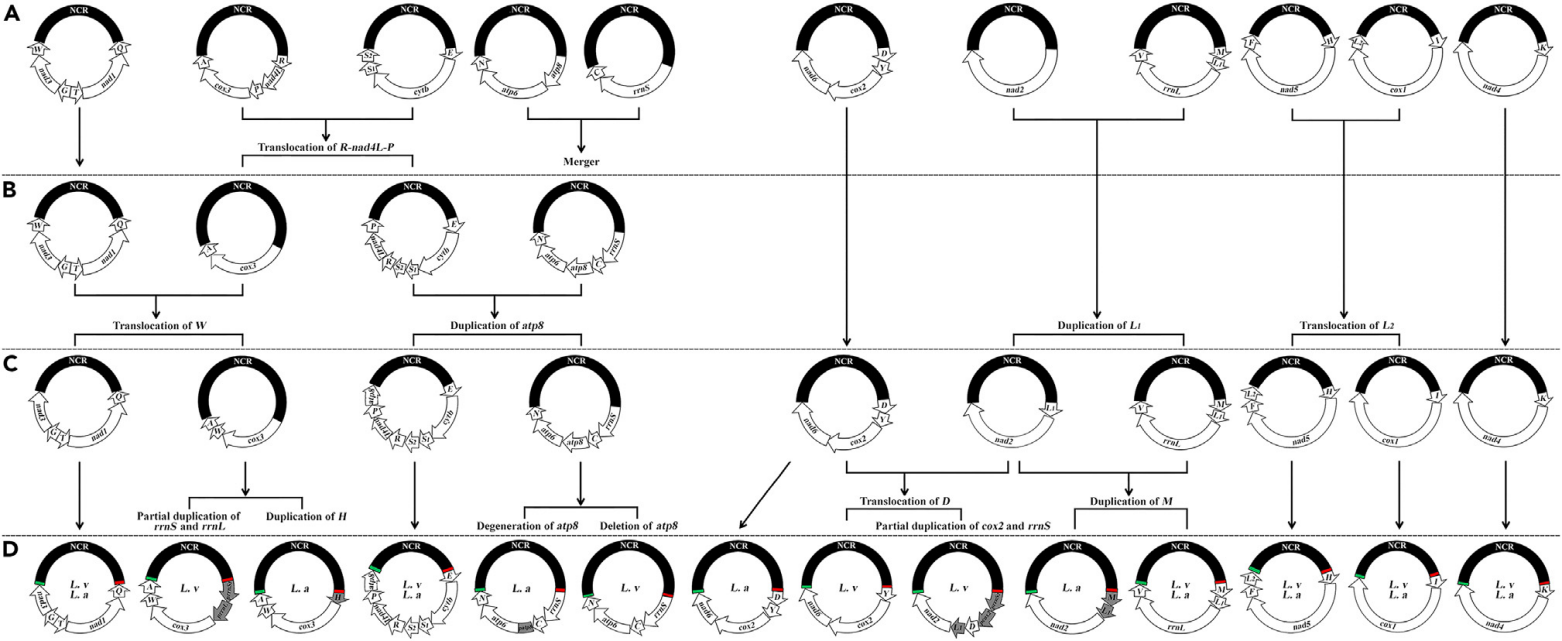
Inferred recombination events that occurred in the evolution of *Linognathus* species from the most recent common ancestor (MRCA) of sucking lice (Anoplura) (A) Mitochondrial (mt) minichromosomes of the MRCA of sucking lice.^11^ (B) Inferred inter-minichromosomal recombination events. (C) Minichromosomes of the MRCA of *Linognathus* spp. (D) Minichromosomes observed in *L. africanus* (*L. a*) and *L. vituli* (*L. v*). See Figure 1 legend for information of mt minichromosomes, genes and large non-coding regions (NCR). Duplicate genes and pseudo genes are shaded in gray. Large NCRs are shaded in black.

The current study reports the complete mt genomes of two *Linognathus* species from the family Linognathidae for the first time. Like the 21 species of sucking lice from other eight families studied previously,^4^^,^^5^^,^^6^^,^^7^^,^^8^^,^^9^^,^^10^^,^^11^^,^^12^^,^^13^^,^^14^^,^^15^^,^^16^ the two *Linognathus* species also have fragmented mt genomes. The *Linognathus* species, however, differ substantially in mt karyotype from other species of sucking lice including *Hoplopleura* species which are most closely related to *Linognathus* species (Figures 4 and S6) and from the inferred most recent common ancestor (MRCA) of sucking lice.^11^ There are five differences in mt karyotype between the *Linognathus* species and the MRCA of sucking lice. First, the *Linognathus* species have 10 minichromosomes, which are one less than the MRCA of sucking lice (Figures 1 and 2; Tables 1 and 2).^11^ Second, as detailed previously, the two *Linognathus* species have four duplicate tRNA genes and five pseudo genes in total, none of which is in the MRCA of sucking lice.^11^ Third, *rrnS* and *atp6*, which are on two different minichromosomes in the MRCA of sucking lice, are on the same minichromosome in both *Linognathus* species. Fourth, *trnL*_*2*_ and *trnW* of both *Linognathus* species and *trnD* of *L. vituli* are on different minichromosomes relative to their corresponding tRNA genes of the MRCA of sucking lice. Fifth, *cytb*, *nad4L*, and *atp8*, which are on three different minichromosomes in the MRCA of sucking lice, are on the same minichromosome in both *Linognathus* species. The separation of *atp8* and *atp6* into different minichromosomes is unique for the *Linognathus* species; all other sucking louse species sequenced to date have the *atp8*-*atp6* gene cluster on a minichromosome of their own and this is inferred to be ancestral to all sucking lice.^4^^,^^5^^,^^6^^,^^7^^,^^8^^,^^9^^,^^10^^,^^11^^,^^12^^,^^13^^,^^14^^,^^15^^,^^16^ Indeed, *atp8*-*atp6* gene cluster is present in the mt genomes of most animals.^21^^,^^22^^,^^23^ Furthermore, a pseudo *atp8* gene is present in *L. africanus* (Figure 2). Apparently, duplication of *atp8* occurred in the MRCA of the two *Linognathus* species, thus adding a copy of *atp8* to the minichromosome that contained *cytb*, *nad4L*, and five tRNA genes (Figure 5). Subsequently, the original copy of *atp8* upstream of *atp6* degenerated to become a pseudo *atp8* in *L. africanus* but was deleted in *L. vituli* (Figure 5).

### Mitochondrial minichromosome recombination is highly active in *Linognathus* lice

As introduced, evidence for recombination between mt minichromosomes has been found in many sucking louse species.^4^^,^^5^^,^^6^^,^^7^^,^^8^^,^^9^^,^^10^^,^^11^^,^^12^^,^^14^^,^^16^ Identical sequences up to 133 bp long shared between genes on different minichromosomes were reported as unequivocal evidence for inter-minichromosomal recombination.^4^^,^^5^^,^^6^^,^^8^^,^^9^^,^^10^^,^^11^^,^^12^^,^^14^^,^^16^ Evidence for both homologous recombination and non-homologous recombination between mt minichromosomes is found in the human body louse based on the observation of eight types of chimeric mt minichromosomes.^18^ The *Linognathus* lice investigated in the current study provided different type of evidence for inter-minichromosomes recombination not seen previously in other sucking lice. Four observations lead us to suggest that inter-minichromosomal recombination is highly active in *Linognathus* lice. First, in the lineage leading to *Linognathus* lice, two ancestral minichromosomes of sucking lice (*rrnS-trnC* and *atp8-atp6-trnN*) merged as one; five genes, i.e., *trnL*_*2*_, *trnR-nad4L-trnP*, and *trnW*, translocated from their inferred ancestral minichromosomes to other minichromosomes; and *atp8* and *trnL*_*1*_ were duplicated leading to two copies of each gene on different minichromosomes (Figure 5). Second, after the divergence of *L. africanus* from *L. vituli*, *trnH*, and *trnM* were duplicated in *L. africanus* leading to two copies of both *trnH* and *trnM* on different minichromosomes (Figures 2 and 5). Third, after the divergence of *L. vituli* from *L. africanus*, portions of *cox2*, *rrnL*, and *rrnS* were duplicated in *L. vituli*, leading to a pseudo *cox2* gene, two pseudo *rrnS* genes and a pseudo *rrnL* gene on different minichromosomes from those of their corresponding full-length genes (Figures 1 and 5). Fourth, in both *Linognathus* species, the duplicate genes and pseudo genes (except pseudo *atp8* gene) are identical or 99.5% similar in sequence to their corresponding full-length genes or a portion of their corresponding full-length genes (Figures S1–S3). There is no evidence for mt minichromosome split in *Linognathus* lice. Selection may maintain sequence similarities but cannot produce duplicate genes or pseudo genes in the first place. Thus, recent homologous recombination between minichromosomes is probably the most plausible explanation for such high sequence similarity between the duplicate tRNA genes and between the pseudo genes and their corresponding full-length genes. The observation of such high sequence similarity simultaneously in four duplicate tRNA genes and four pseudo genes indicates rather frequent homologous recombination between mt minichromosomes in the two *Linognathus* species.

### Minichromosomal locations upstream of *cox3* and *nad2* are recombination hotspots in *Linognathus* lice

Ten of the 11 mt minichromosomes of the MRCA of sucking lice^11^ were involved in one or more recombination events in the evolution of *Linognathus* species, either minichromosome merger or tRNA translocation or gene duplication (Figure 5). *trnK-nad4* minichromosome is the only one that is not involved in any recombination events and remains unchanged in gene content and gene order (Figure 5). Of all the mt minichromosomal locations, the location upstream of *cox3* and the location upstream of *nad2* had the most frequent recombination events (Figure 5). The gene cluster *R-nad4L-P* upstream of *cox3* was translocated to *trnE-cytb-trnS*_*1*_*-trnS*_*2*_ minichromosome in the lineage leading to *Linognathus* species (Figure 5). The translocation of *trnR-nad4L-trnP* would require at least one recombination event between *trnR-nad4L-trnP-cox3-trnA* minichromosome and *trnE-cytb-trnS*_*1*_*-trnS*_*2*_ minichromosome (Figure 5), both ancestral to sucking lice.^11^ Subsequently, two pseudo genes, *prrnS*_*1*_ and *prrnL*, were inserted upstream of *cox3* in *L. vituli*, whereas in *L. africanus* a duplicate copy of *trnH* gene was inserted in this location (Figures 1, 2, and 5). The insertion of *prrnS*_*1*_, *prrnL*, and duplicate *trnH* would require at least three recombination events as *rrnS*, *rrnL*, and *trnH* were on three different minichromosomes in the MRCA of sucking lice (Figure 5).^11^ There was no gene upstream of *nad2* in the MRCA of sucking lice (Figure 5). In the lineage leading to *Linognathus* species, a duplicate copy of *trnL*_*1*_ gene was inserted upstream of *nad2* (Figures 1 and 2). The insertion of duplicate *trnL*_*1*_ would require one recombination event between *nad2* minichromosome and *trnM-trnL*_*1*_*-rrnL-trnV* minichromosome, both ancestral to sucking lice (Figure 5).^11^ Subsequently, *trnD* was translocated upstream of *nad2-trnL*_*1*_ and two pseudo genes, *prrnS*_*2*_ and *pcox2*, were inserted upstream of *nad2-trnL*_*1*_*-trnD* in *L. vituli*; whereas in *L. africanus* a duplicate copy of *trnM* gene was inserted upstream of *nad2-trnL*_*1*_ (Figures 1 and 2). The translocation of *trnD* and the insertion of *prrnS*_*2*_, *pcox2*, and duplicate *trnM* would require at least three recombination events as *trnD*, *rrnS*, *cox2*, and *trnM* were on three different minichromosomes in the MRCA of sucking lice, or possibly four recombination events as *trnD* translocation and *pcox2* insertion might occur separately although *trnD* and *cox2* were on the same minichromosome in the MRCA of sucking lice (Figure 5).^11^

As discussed previously, all of the duplicate genes and pseudo genes upstream of *cox3* and *nad2* in both *Linognathus* species have identical or near identical sequences as their corresponding original copy of genes or a portion of their corresponding full-length genes (Figures S1–S3). Retention of multiple duplicate or pseudo genes in the highly compact mt genomes of animals is uncommon^1^; redundant genes often become degenerated and deleted. Recent homologous recombination that involved the minichromosomal locations upstream of *cox3* and *nad2* is probably the most plausible explanation for the extremely high sequence similarity between the duplicate genes and their corresponding original copy of genes, and between the pseudo genes and their corresponding full-length genes in the *Linognathus* species.

### Limitations of the study

Sucking lice (Anoplura) parasitize members of 12 of the 29 mammalian orders and ∼840 mammalian species.^19^ To date, more than 540 species of sucking lice have been described and assigned to 50 genera in 15 families.^24^^,^^25^ The genus *Linognathus* has 51 species that parasitize a range of mammals in four families (Bovidae, Cervidae, Giraffidae, and Canidae) of two orders (Artiodactyla and Carnivora).^24^^,^^26^ The hosts of the two *Linognathus* species we sequenced in the current study are both in the family Bovidae. To verify the findings of the current study, *Linognathus* species that parasitize mammals in the Cervidae, Giraffidae, and Canidae should be sequenced and compared in future studies. Furthermore, the current study used a comparative mt genomics approach, to understand minichromosomal recombination in the mt genomes of parasitic lice, biochemical experimental approaches are also needed.

## STAR★Methods

### Key resources table

**Table:** 

REAGENT or RESOURCE	SOURCE	IDENTIFIER
**Chemicals, peptides, and recombinant proteins**		

EmeraldAmp Max HS PCR Master Mix (2X Premix)	TaKaRa	Cat# RR330; Lot# AM11628A
Proteinase K	TRANS	Code# GE201-01; Lot# R10224
RNase A	TRANS	Code# GE101-01; Lot# P10705

**Critical commercial assays**		

QIAamp® DNA Micro Kit	QIAGEN	Cat. No. 56304

**Deposited data**		

Raw data of newly sequenced *Linognathus vituli*	Sequence Read Archive data (SRA)	PRJNA982721
Raw data of newly sequenced *Linognathus africanus*	SRA	PRJNA983060
Newly sequenced *Linognathus vituli* mitochondrial (mt) genome data	GenBank	OL677823-32
Newly sequenced *Linognathus africanus* mt genome data	GenBank	OP948897-906

**Software and algorithms**		

Trimmomatic v.0.36	Bolger et al., 2014^27^	https://doi.org/10.1093/bioinformatics/btu170
Geneious 11.1.5	Kearse et al., 2012^28^	https://doi.org/10.1093/bioinformatics/bts199
MITOS WebServer	Bernt et al., 2013^29^	http://mitos.bioinf.uni-leipzig.de/index.py
ARWEN	Laslett and Canbäck, 2008^30^	http://130.235.244.92/ARWEN/
tRNAscan-SE	Lowe and Chan, 2016^31^	http://lowelab.ucsc.edu/tRNAscan-SE/
Wordmatch	Rice et al., 2000^32^	https://www.bioinformatics.nl/cgi-bin/emboss/wordmatch
MAFFT version 7.0	Katoh and Standley, 2016^33^	https://doi.org/10.1093/bioinformatics/btw108
Gblocks 0.91b	Talavera and Castresana, 2007^34^	http://phylogeny.lirmm.fr/phylo_cgi/one_task.cgi?task_type=gblocks
MrBayes 3.2.6	Ronquist et al., 2012^35^	https://doi.org/10.1093/sysbio/sys029
ProtTest 3.4	Darriba et al., 2011^36^	https://doi.org/10.1093/bioinformatics/btr088
PhyML 3.0	Guindon et al., 2010^37^	https://doi.org/10.1093/sysbio/syq010

**Other**		

The inferred most recent common ancestor (MRCA) of sucking lice (Anoplura)	Shao et al., 2017^11^	https://doi.org/10.1093/gbe/evx007

### Resource availability

#### Lead contact

DNA sequence information is publicly available from the NCBI and EBI databases. Further information and requests for resources should be directed to and will be fulfilled by the lead contact, Prof Guo-Hua Liu (liuguohua5202008@163.com).

#### Materials availability

This study did not generate new unique reagents.

### Experimental model and study participant details

#### Louse samples

All procedures involving animals in the present study were approved and this study was approved by the Animal Ethics Committee of Hunan Agricultural University (No. 201703386). The adult cattle louse *L. vituli* samples were collected from the body surface of a bull *Bos taurus* slaughtered in Hunan province, China. The adult goat louse *L. africanus* samples were collected from the body surface of a healthy male goat *Capra hircus* in Khyber Pakhtunkhwa province, Pakistan. The animals’ entire body was inspected visually for the presence of lice for three minutes. All louse specimens were removed with the aid of a forceps and stored in centrifuge tubes. Morphological analysis of lice were performed using a stereoscopic microscope (Nikon SMZ18, Tokyo, Japan).^25^ Cattle louse is 2.0–3.0 mm long, and goat louse is 2.1–2.9 mm in length. In addition, the sex was identified based on morphological characteristics: male with basal apodeme slender and pseudopenis elongate or poorly sclerotized; parameres well developed; female with subgenital plate variously shaped, gonopods VIll well developed; gonopod IX well developed and prolonged posteriorly, with either a spiniform genital seta or pointed apical process; spermatheca not strongly sclerotized. All louse specimens were then washed five times in physiological saline solution, and stored in 100% (v/v) ethanol at −40°C.

### Method details

#### DNA extraction

For each louse species, total genomic DNA was extracted from 50 individual lice (25 females and 25 males) using QIAamp® DNA Micro Kit (QIAGEN, Hilden, Germany) according to the manufacturer’s recommendations. The species identity of each specimen was verified by sequencing regions of mt *cox1* and *rrnS* genes.^11^^,^^38^ The *cox1* and *rrnS* gene sequences of our *L. vituli* specimens have 97% and 100% similarity with that of *L. vituli* from *B. taurus* in Australia (accession numbers HM241900 and HM241899, respectively). The *cox1* gene sequences of our *L. africanus* specimens have 99.7% similarity with that of *L. africanus* from *C. hircus* in Peru (accession no. EU375760).

#### Sequencing and assembling

Genomic DNA concentration was determined using Qubit 4.0 (Invitrogen, Carlsbad, USA); DNA integrity was analysed with agarose-gel electrophoresis. Genomic DNA library (530-bp inserts) was constructed for high-throughput sequencing with Miseq (Illumina, San Diego, CA, USA); paired-end raw reads (300 bp each) were exported in FASTQ format. Raw reads were trimmed and filtered using Trimmomatic v.0.36.^27^ For each *Linognathus* louse species, 2Gb of high-quality clean reads were obtained after removing adaptor sequences, highly redundant sequences, reads containing more than 10% ‘N’ (‘N’ representing ambiguous bases in reads), and reads containing more than 50% bases with Q-value ≤20. We used the Map-to-Reference(s) option in Geneious 11.1.5^28^ to assemble mt genomes. The *cox1* and *rrnS* sequences of the two *Linognathus* lice were used respectively as the initial references to assemble the Miseq sequence reads. The assembly parameters were minimum overlap identity 99% and minimum overlap 200 bp. The assemblies were iterated until *cox1* and *rrnS* minichromosomes were assembled in full length for the gene-containing region and from each end of this region, extended ∼500 bp into the large non-coding regions (NCRs). Previous studies showed that the large NCRs are highly conserved among the mt minichromosomes of each sucking louse species^11^^,^^12^^,^^13^ or chewing louse species.^39^ The conserved large NCR sequences were identified between the *cox1* and *rrnS* minichromosomes and were used as references to align the Miseq sequence reads. This allowed us to extract sequence reads derived from the two ends of the gene-containing regions of all other mt minichromosomes. We then assembled these minichromosomes individually in full length for the gene-containing region and extended the contigs into the large NCRs as we did above for *cox1* and *rrnS* minichromosomes.

#### Verification and sequencing of individual mitochondrial minichromosomes

The size and circular structure of each mt minichromosome of *L. vituli* and *L. africanus* were verified by PCR using specific primers targeting gene-containing regions (Tables S1 and S2). The forward primer and reverse primer in each pair were next to each other with a small gap less than 10 bp. PCR with these primers amplified each circular minichromosome in full length. To obtain full-length sequence of the large NCRs of each minichromosome, the PCR amplicons were also sequenced with Miseq as described above. Thus, the full-length sequences of both gene-containing region and large NCRs of each minichromosome were obtained. In addition, to confirm the presence of pseudo *atp8* gene between *trnC* and *atp6* in *L. africanus*, PCR primers were designed to amplify the region between *rrnS* and *atp6*, and the amplicon was sequenced using Sanger method by the Sangon Company (Shanghai).

#### Mitochondrial genome annotation

The assembled mt genomes were annotated initially with MITOS WebServer (http://mitos.bioinf.uni-leipzig.de/index.py).^29^ tRNA genes annotated by MITOS were verified using ARWEN^30^ and tRNAscan-SE^31^; the inferred secondary structure of each tRNA was checked manually. Protein-coding genes annotated by MITOS were verified using ORFfinder (https://www.ncbi.nlm.nih.gov/orffinder/) and BLAST searches of GenBank (https://blast.ncbi.nlm.nih.gov/Blast.cgi). The start and stop codon positions of each protein-coding gene were checked manually and determined by minimizing intergenic space and overlap between neighbour genes. rRNA genes annotated by MITOS were also verified by BLAST searches of GenBank and by sequence alignment and comparison with published species of sucking lice. The start and stop positions of each rRNA gene were checked manually and determined by minimizing intergenic spaces and overlap between neighbour genes. Identical sequences shared between genes were searched with Wordmatch.^32^ Sequence alignment was made with MAFFT version 7.122.^33^ The circular maps of mt genomes were drawn with Microsoft PowerPoint v.2021.

#### Phylogenetic analysis

Amino acid sequences were deduced from the nucleotide sequences of eight mt protein-coding genes common for 21 species of sucking lice and the elephant louse (Table S3). The deduced amino acid sequences were aligned individually using MAFFT 7.122^33^ and concatenated to form a single dataset; ambiguously aligned regions were excluded using Gblocks 0.91b using default parameters.^34^ Bayesian phylogenetic analyses were conducted using MrBayes 3.2.6.^35^ The most suitable model (MtArt) of evolution was selected by ProtTest 3.4^36^ using the default setting based on the Akaike information criterion (AIC). MtArt model, however, could not be implemented in the current version of MrBayes, thus the best scoring alternative model MtREV was used. Four independent Markov chains were run simultaneously for 1,000,000 metropolis coupled MCMC generations, sampling a tree every 100 generations. The first 2,500 trees represented burn-in, and the remaining trees were tested for stability of likelihood values and used to compute Bayesian posterior probability (Bpp). We assumed that stationarity had been reached when the estimated sample size (ESS) was greater than 100, the potential scale reduction factor (PSRF) approached 1.0 and the average standard deviation of split frequencies (ASDSF) was <0.01. Meanwhile, phylogenetic analyses were conducted with maximum-likelihood method (ML) using PhyML 3.0^37^ with a BioNJ starting tree, and tree topology search was set from the subtree pruning and regrafting (SPR) method. Bootstrap value (Bv) was calculated using 100 bootstrap replicates. Phylogenetic trees were drawn using FigTree v.1.42.

### Quantification and statistical analysis

DNA concentration of each sample was determined using the Qubit 4.0 system (Thermo Fisher Scientific, Waltham, MA, USA). Total DNA sequencing was performed by Miseq (Illumina, San Diego, CA, USA) to produce 2 × 300 bp paired-end reads and raw data were recorded in FASTQ format. The raw reads were filtered to remove containing adaptor sequences and low-quality reads (the ‘N’ percent of one end >5%, average quality score Q < 20 and length <75 bp after trimming) using Trimmomatic v.0.36.^27^

The phylogenetic analysis was carried out as described above. Datasets were processed by the program MAFFT 7.122^33^ with “E-INS-I” strategy, and Gblocks 0.91b^34^ with less stringent selection. Phylogenetic trees were constructed using the Bayesian and ML methods. The software MrBayes 3.2.6^35^ was used for Bayesian analysis. Bpp had statistical significance when the ESS was >100, the PSRF approached 1.0 and the ASDSF was <0.01. ML analysis was performed by the software PhyML 3.0,^37^ and Bv was calculated using 100 bootstrap replicates. The statistical details were showed in the results, Figures 4 and S6.

### Additional resources

This study has not generated or contributed to a new website/forum, and is not a part of a clinical trial.

## Data Availability

• The raw data and assembled mt genomes generated in the present study have been deposited in NCBI database under the following accession numbers: PRJNA982721 and PRJNA983060, and OL677823-32 and OP948897-906. • This paper does not report original code. • Any additional information required to reanalyze the data reported in this paper is available from the lead contact upon request.
