# Supplementary material for: The fragmented mitochondrial genomes of two Linognathus lice reveal active minichromosomal recombination and recombination hotspots
Source: iScience. 2023 Jul 16;26(8):107351. doi: 10.1016/j.isci.2023.107351 (PMC10382929; doi:10.1016/j.isci.2023.107351)
Supplement: Document S1. Figures S1–S6 and Tables S1–S3 [file mmc1.pdf]

iScience, Volume *26*

Supplemental information

The fragmented mitochondrial genomes of two *Linognathus* lice reveal active minichromosomal recombination and recombination hotspots

Yi-Tian Fu, Renfu Shao, Suleman, Wei Wang, Hui-Mei Wang, and Guo-Hua Liu

Supplemental information

Table S1 PCR primers used to amplify the mitochondrial minichromosomes of the cattle louse, *Linognathus vituli*, related to Figure 1.

Primer	Sequence (5’ to 3’)	Minichromosome
*cytb*F	ATACTCTGACAAGACTGGTTCGTGA	*E-cytb-S*$_1$*-S*$_2$*-R-nad4L-P-atp8*
*cytb*R	CTTACTTCACGAACCAGTCTTGTCA	*E-cytb-S*$_1$*-S*$_2$*-R-nad4L-P-atp8*
*rrnS*F	TACACGAACATCTTACCCCTCTTT	*rrnS-C-atp6-N*
*rrnS*R	AGAGGGGTAAGATGTTCGTGTAGC	*rrnS-C-atp6-N*
*cox1*F	TACCTACTGTTTGGGACCTGATGTG	*I-cox1*
*cox1*R	CACATCAGGTCCCAAACAGTAGGTA	*I-cox1*
*cox2*F	ATCAAACCCTCCTCACACCAACAAA	*Y-cox2-nad6*
*cox2*R	GCATCTATCTTTAGCCCTATTCTGG	*Y-cox2-nad6*
*cox3*F	TGGGTTTATTCTGTTTATCCTCTCG	*prrnS*$_1$*-prrnL-cox3-W-A*
*cox3*R	TGATAAGTTCTCTCCCGCACTACAT	*prrnS*$_1$*-prrnL-cox3-W-A*
*nad3*F	TGACTATCGTTATCAGCCTCATCCT	*Q-nad1-T-G-nad3*
*nad3*R	GATACCTCTGTAGTTTACCCGAATA	*Q-nad1-T-G-nad3*
*nad2*F	ACTTATCAGGTTATCGGGAGTAGCA	*prrnS*$_2$*-pcox2-D-L*$_1$*-nad2*
*nad2*R	AGTCATTGCTACTCCCGATAACCTG	*prrnS*$_2$*-pcox2-D-L*$_1$*-nad2*
*nad4*F	GACTCCCTCATTATGGGTAACACTA	*K-nad4*
*nad4*R	TAGTGTTACCCATAATGAGGGAGTC	*K-nad4*
*nad5*F	TTGCCTACTCAACCCTATCCCACAT	*H-nad5-F-L*$_2$
*nad5*R	TAATGTGGGATAGGGTTGAGTAGGC	*H-nad5-F-L*$_2$
*rrnL*F	TAGTACGAAAGGACACGGGGAAGGG	*M-L*$_1$*-rrnL-V*
*rrnL*R	AATCAAATCCCTTCCCCGTGTCCTT	*M-L*$_1$*-rrnL-V*

Table S2 PCR primers used to amplify the mitochondrial minichromosomes of the goat louse, *Linognathus africanus*, related to Figure 2.

Primer	Sequence (5’ to 3’)	Minichromosome
*cytb*F	GGGGTTGATTGATTCGGGTTTGT	*E-cytb-*S_1*-*S_2*-R-nad4L-P-atp8*
*cytb*R	AACCCTTCTAAATGCTAAGTCCA	*E-cytb-*S_1*-*S_2*-R-nad4L-P-atp8*
*rrnS*F	AACACTCTTGTGAAATGGTAACT	*rrnS-C-atp6-N*
*rrnS*R	ACAAGAGTGTTTTGAACCCCCAT	*rrnS-C-atp6-N*
*cox1*F	GAAGAGTCAGGTAAGAAGGAGAT	*I-cox1*
*cox1*R	ATCTCCTTCTTACCTGACTCTTC	*I-cox1*
*cox2*F	GTCAATGCTCTGAAATGTGTGGG	*D-Y-cox2-nad6*
*cox2*R	TAGCCCCACACATTTCAGAGCAT	*D-Y-cox2-nad6*
*cox3*F	CTCACACTAAGGAGGTTGTTGCT	*H-cox3-W-A*
*cox3*R	TCCTTAGTGTGAGCCCCTTGATA	*H-cox3-W-A*
*nad1*F	CAACAACGGCAACTTTATTAGGG	*Q-nad1-T -G-nad3*
*nad1*R	GAATTTCTCAAACCCGAGTAGGC	*Q-nad1-T -G-nad3*
*nad2*F	CCCTTCTAACCATTATTCCGTGA	*M-*L_1*-nad2*
*nad2*R	TCACGGAATAATGGTTAGAAGGG	*M-*L_1*-nad2*
*nad4*F	CCAATGGTTTATTTGATTCCGAC	*K-nad4*
*nad4*R	ATAAACAACCCCAAACCCGTCCC	*K-nad4*
*nad5*F	GCATACAGGTCTCGGTTAGCGTT	*H-nad5-F-*L_2
*nad5*R	AACGCTAACCGAGACCTGTATGC	*H-nad5-F-*L_2
*rrnL*F	AGATGAAGGAGAGGGACGAGAAG	*M-*L_1*-rrnL-V*
*rrnL*R	CTCTCCTTCATCTGAGCATTTTC	*M-*L_1*-rrnL-V*

Table S3 The sucking lice included in phylogenetic analyses in this study, related to Figure 5.

Species	Host	GenBank accession number
Antarctophthirus carlinii	Seal	MW803073-81
Antarctophthirus microchir	Sea lion	MW803082-93
Haematopinus apri	Wild pig	KC814611-19
Haematopinus asini	Horse	KF939318, KF939322, KF939324, KF939326, KJ434034-38
Haematopinus suis	Domestic pig	KC814602-10
Haematopinus tuberculatus	Buffalo	ON416547-56
Hoplopleura kitti	Rat	KJ648933-43
Hoplopleura sp.	Rat	MT792483-94
Lepidophthirus macrorhini	Elephant seal	MW803094-104
Microthoracius praelongiceps	Guanaco	KX090378-KX090389
Pediculus humanus capitis	Human	JX080388-407
Pediculus humanus humanus	Human	FJ499473-90
Pediculus schaeffi	Chimpanzee	KC241882-97, KR706168-69
Pedicinus badii	Monkey	MT721726-37
Pedicinus obtutas	Monkey	MT792495–506
Pthirus gorillae	Gorilla	MW803115-131
Pthirus pubis	Human	JQ976018, MT721740, HM241895-8, EU219987-95
Polyplax asiatica	Rat	KF647751-61
Polyplax reclinata	Shrew	MW291451-61
Polyplax spinulosa	Rat	KF647762-72
Linognathus africanus	Goat	OP948897-906
Linognathus vituli	Cattle	OL677823-32

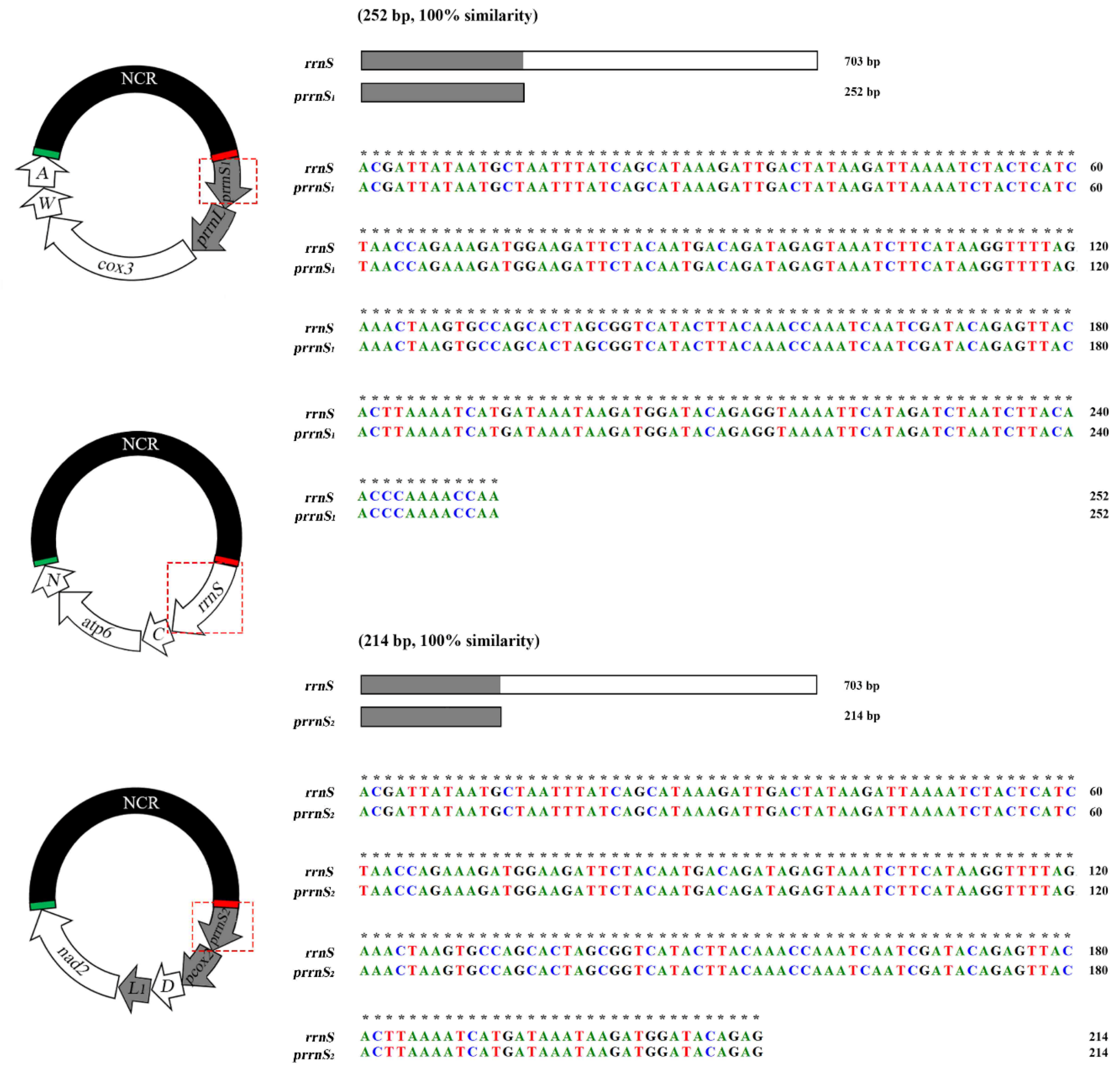

Figure S1. Sequence alignments, lengths and similarities of pseudo genes (*prrnS$_1$* and *prrnS$_2$*) with their corresponding full-length genes in the cattle louse *Linognathus vituli*, related to Figure 1. Dotted boxes in red indicate pseudo genes (*prrnS$_1$* and *prrnS$_2$*) and corresponding full-length *rrnS* gene. Rectangle in gray indicate the location of the same nucleotide sequence of the pseudo genes (*prrnS$_1$* and *prrnS$_2$*) and corresponding full-length *rrnS* gene. Gene name and transcription orientation are indicated in the coding region; non-coding regions (NCR) are in black. See Figure 1 legend for gene name abbreviation.

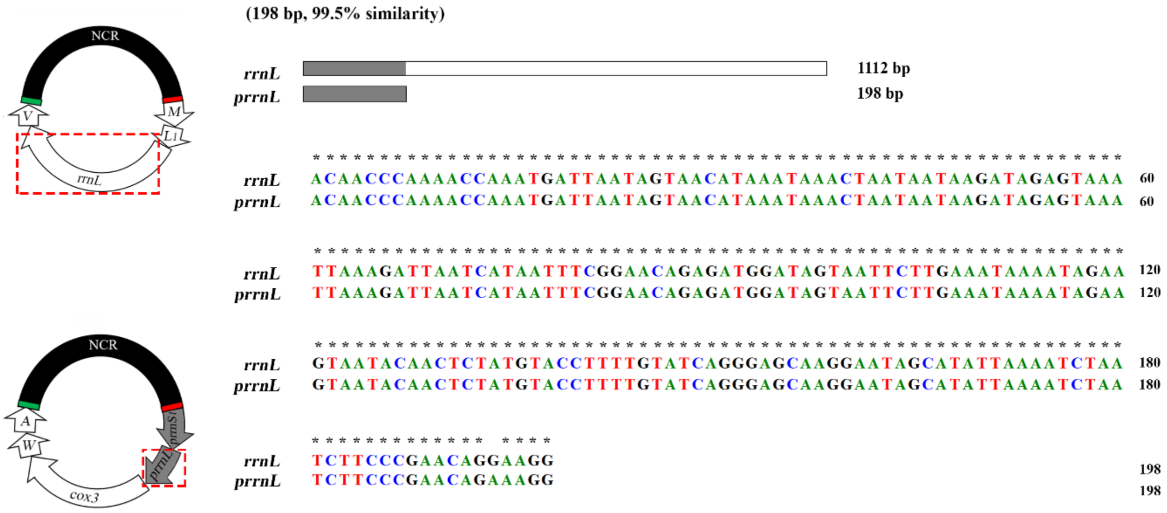

Figure S2. Sequence alignment, length and similarity of pseudo-*rrnL* gene with its corresponding full-length gene in the cattle louse *Linognathus vituli*, related to Figure 1. Dotted boxes in red indicate pseudo-*rrnL* gene and corresponding full-length *rrnL* gene. Rectangle in gray indicate the location of the same nucleotide sequence of the pseudo-*rrnL* gene and corresponding full-length *rrnL* gene. Gene name and transcription orientation are indicated in the coding region; non-coding regions (NCR) are in black. See Figure 1 legend for gene name abbreviation.

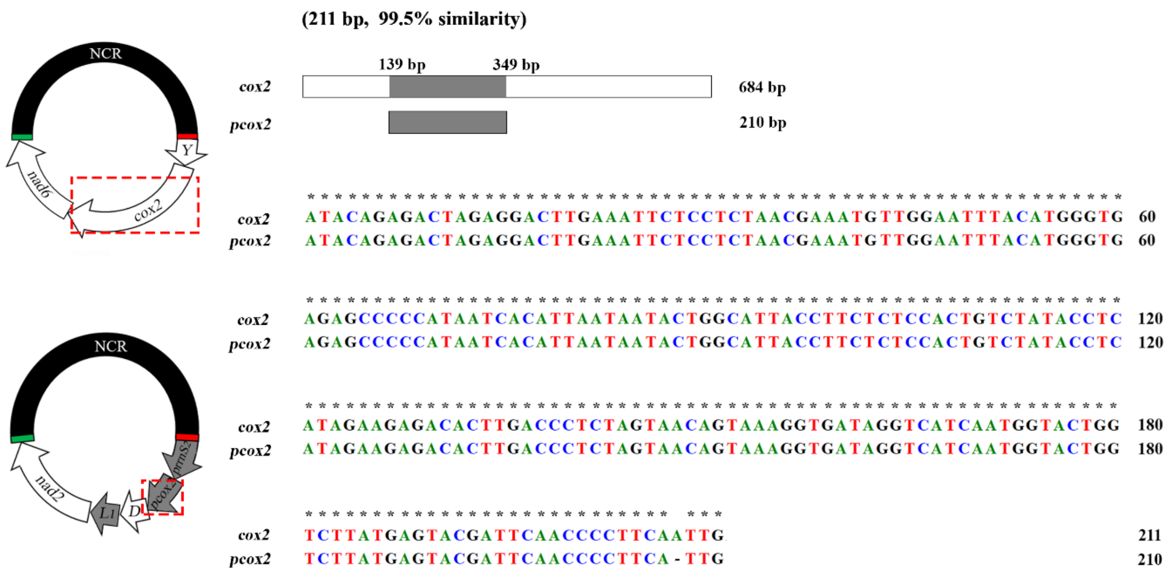

Figure S3. Sequence alignment, length and similarity of pseudo-*cox2* gene with its corresponding full-length gene in the cattle louse *Linognathus vituli*, related to Figure 1. Dotted boxes in red indicate pseudo-*cox2* gene and corresponding full-length *cox2* gene. Rectangle in gray indicate the location of the same nucleotide sequence of the pseudo-*cox2* gene and corresponding full-length *cox2* gene. Gene name and transcription orientation are indicated in the coding region; non-coding regions (NCR) are in black. See Figure 1 legend for gene name abbreviation.

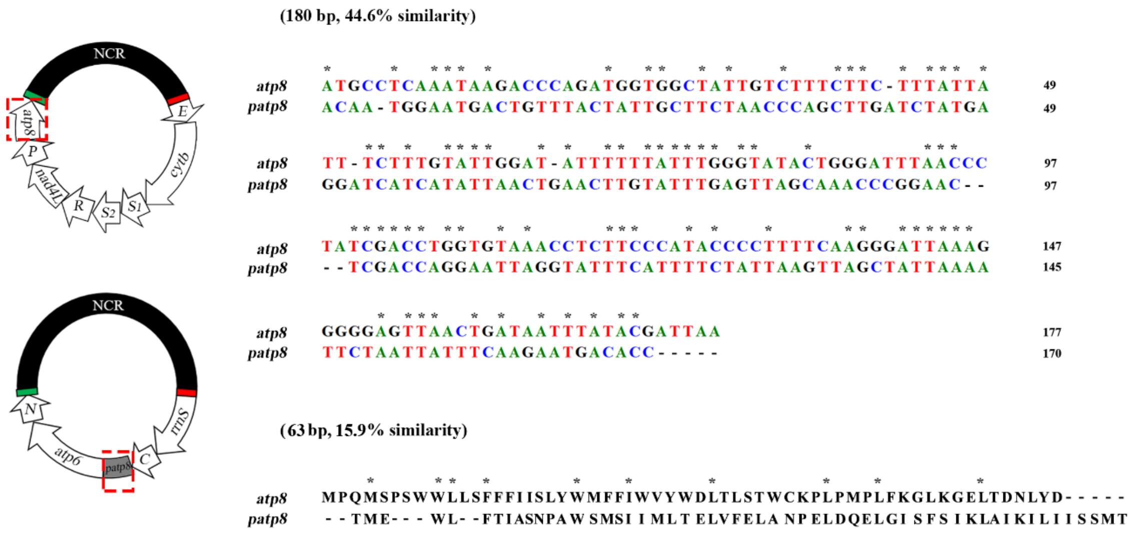

Figure S4. Nucleotide and amino acid sequences alignments, lengths and similarities of pseudo-*atp8* gene with its corresponding full-length gene in the goat louse *Linognathus africanus*, related to Figure 2. Dotted boxes in red indicate pseudo-*atp8* gene and corresponding full-length *atp8* gene. Rectangle in gray indicate the location of the same nucleotide sequence of the pseudo-*atp8* gene and corresponding full-length *atp8* gene. Gene name and transcription orientation are indicated in the coding region; non-coding regions (NCR) are in black. See Figure 1 legend for gene name abbreviation. The pseudo-*atp8* can form an open reading frame but does not have a start codon, nor a stop codon, nor the highly conserved amino acid sequence MPQ at the beginning of the open reading frame.

atp8 gene (186 bp, 51.6% similarity)

```
             *********** ** **  * *********   ** ********  * ** *   * * *
L. vituli    ATGCCTCAAATGAGTCCATGGTGGTGGCTACCTTCATTCTTCTTAGTGATCTTAGTCTTT    60
L. africanus ATGCCTCAAATAAGACCCAGATGGTGGCTATTGTCTTTCTTCTTTATTATTTCTTTGTAT    60

                ** *** *    ** ******** *****     ***   *   ** *      ** 
L. vituli    ATAATCTTTATATCATGAGTATACTGAGATTTTCAATTATTTTCAGTGTATTTTATTAAG    120
L. africanus TGGATATTTTTTATTTGGGTATACTGGGATTTAACCCTATCGACCTGGTGT------AAA    114

              *      ** * *  ** *    **    * *  *    * *  **   ***  *
L. vituli    GCAACCGACAGAACGGTTCTGGTAGGGCGGAGGAAGCCTAATTCGACCCCTAACCTTCTT    180
L. africanus CCTCTTCCCATACCCCTTTTCAAGGGATTAAAGGGG---GAGTTAACTGATAATTTATAC    171

                ***
L. vituli    AGGTAA                                                          186
L. africanus GATTAA                                                          177
```

(61 bp, 42.6% similarity)

```
             ****** *** ***  *     *  *****  *      *        *  *   * **
L. vituli    MPQMSPWWWLPSFFLVILVFMIFMSWVYWDFQLFSVYFIKATDSTVLVGRSKPNSTPNLLS    61
L. africanus MPQMSPSWWLLSFFFIISLYWMFFIWVYWDLTLS--TWCKPLPMPLFKG-LKGELTDNLYD    58
```

Figure S5. Nucleotide and amino acid sequence alignments, lengths and similarities of *atp8* gene between the two *Linognathus* lice, related to Figures 1 and 2. The *atp8* genes of both species have the start codon ATG, the stop codon TAA, and the highly conserved amino acid sequence MPQ at the beginning of the open reading frame.

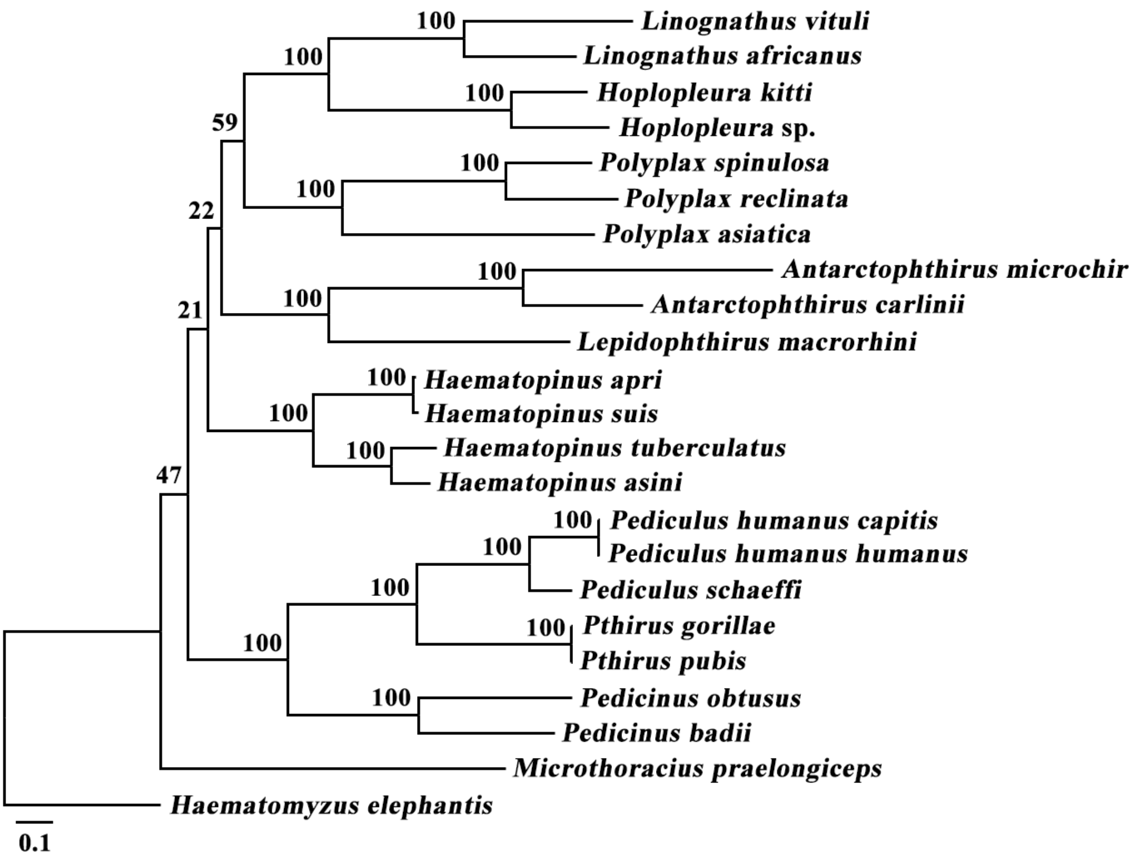

Figure S6. Phylogenetic relationships among 21 species of sucking lice inferred by maximum likelihood method of deduced amino acid sequences of eight mitochondrial proteins, related to Figure 4. The elephant louse, *Haematomyzus elephantis*, was used as the outgroup. Bootstrap support values (%) are indicated at nodes.
